# Micromechanics Modeling on Mechanical Properties in Mg Alloys with Bimodal Grain Size Distribution

**DOI:** 10.3390/nano14221807

**Published:** 2024-11-11

**Authors:** Shaojie Li, Jianfeng Jin, Hao Sun, Yongbo Wang, Yuping Ren, Mingtao Wang, Gaowu Qin

**Affiliations:** 1 School of Materials Science and Engineering, Northeastern University, Shenyang 110819, China; lisj_neu@163.com (S.L.); sunhao1230224@163.com (H.S.); 15702451373@163.com (Y.W.); renyp@atm.neu.edu.cn (Y.R.); wangmingtao@mail.neu.edu.cn (M.W.); 2 Institute of Materials Intelligent Technology, Liaoning Academy of Materials, Shenyang 110167, China; 3 Institute for Strategic Materials and Components, Shenyang University of Chemical Technology, Shenyang 110142, China

**Keywords:** bimodal structure, micromechanics model, strain gradient theory, heterogeneous deformation-induced effect (HDI), mechanical properties

## Abstract

Bimodal grain structure (BGS) Mg alloys containing a high fraction of fine grains (FGs) and a low fraction of coarse grains (CGs) show a good combination of strength and plasticity. Here, taking the ZK60 alloy as an example, the influences of CG size, volume fraction, and texture intensity on mechanical properties and the hetero-deformation-induced (HDI) effect were examined using the Mori–Tanaka mean-field method combined with strain gradient theory of plasticity. The results indicate that the overall mechanical properties decrease with an increase in CG size because the limited HDI effect cannot compensate for the strength and plasticity decrease derived from larger CGs. A higher aspect ratio of CG along the loading direction can weaken the HDI effect and subsequently reduce the overall mechanical properties. Optimal comprehensive mechanical properties can be achieved when the CG volume fraction is approximately 30%. Furthermore, an increasing basal texture intensity in CG results in higher yield strength and lower ultimate tensile strength, while the uniform elongation reaches a maximum value when ~60% of CGs possess hard orientations with Euler angles of (0~30°, 0°, 0°).

## 1. Introduction

Magnesium (Mg) alloys, as the lightest metallic structural materials available today, hold strategic significance for major lightweight projects in aerospace, transportation, and other industrial sectors [1], contributing significantly to lightweight structures, energy savings, emissions reductions, and safe services [2]. However, their further applications are limited by their low strength and relatively poor plasticity. For this reason, heterogeneous structured Mg alloys, with their excellent combination of strength and plasticity, are becoming one of the research hotspots in the field of materials. Their superior mechanical properties are attributed to the synergistic deformation mechanism by the heterogeneous structure, namely the hetero-deformation-induced (HDI) [3,4] strengthening and strain-hardening effect. An alloy with a bimodal grain structure (BGS), consisting of coarse grains (CGs) and fine grains (FGs), is a typical heterogeneous structure. Since Tellkamp et al. [5] and Wang et al. [6] discovered that the BGS 5083 Al alloy and BGS Cu have excellent strength–plasticity synergy, the BGS has been extended to Cu [7,8], Ni [9,10], Fe [11], Al [12,13,14], Mg [15,16,17,18,19,20], and other alloys.

Severe plastic deformation processing is commonly used in practice to achieve incomplete recrystallization in Mg alloys, resulting in BGSs composed of un-dynamically recrystallized (un-DRXed) CG and DRXed FG. The processing includes hot extrusion [15,21,22,23,24,25,26], hard plate rolling [27,28,29], equal channel angular extrusion [30,31], accumulative back extrusion [32], and hot rolling [33]. Some works [34,35,36,37] indicate that increasing the plastic deformation amount and decreasing the deformation rate can facilitate BGS formation. Moreover, for rare-earth-free Mg alloys, BGS prepared at a low extrusion rate [36,37] exhibits a higher texture intensity and lower plasticity. Therefore, unique processing such as hard plate rolling [27,28,29] and two-step extrusion [38] are developed to produce BGSs in rare-earth-free Mg alloys, ensuring weaker basal texture. In rare-earth Mg alloys, higher extrusion speeds facilitate the formation of non-basal textures (typically the <1121> component parallel to the extrusion direction, known as the ‘rare-earth’ texture in certain studies [39,40,41]), leading to enhanced plasticity [42,43,44]. Along with the influence of rare-earth alloying elements in facilitating non-basal slip, this results in BGSs demonstrating outstanding overall properties.

**Figure 1:**
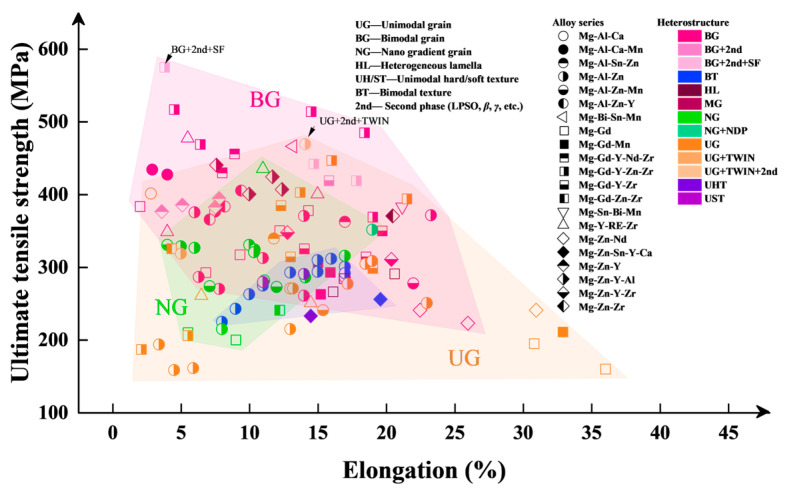
Ashby chart of ultimate tensile strength and elongation for wrought Mg alloys from academic references [45].

Figure 1 shows the relationship between the ultimate tensile strength (UTS) and elongation of homogeneous and heterogeneous structure Mg alloys from academic references [45]. It shows that the UTS of homogeneous Mg alloys is between 150 and 470 MPa and the elongation is between 5% and 35%, while the UTS of heterogeneous alloys is between 200 and 570 MPa, and the elongation is between 3% and 25%. In the BGS Mg alloys, the FG size ranges from a few micrometers [46] to hundreds of nanometers [47], exhibiting a synergistic enhancement of strength and toughness. The CG typically exhibits a grain size of tens of micrometers and contains substructures such as twins [48], stacking faults [49], and precipitates [16,19]. These substructures, together with the hard-oriented CGs, bring about HDI strengthening and strain-hardening effects, resulting in a synergistic effect of strength and plasticity in BGS Mg alloys. For example, the BGS Mg-9Al-1Zn-1Sn alloy [46] achieves a yield strength (YS) of 251 MPa, a UTS of 393 MPa, and a uniform elongation (UEL) of 23%. The BGS WE43 alloy [50] demonstrates a YS of 312 MPa, a UTS of 332 MPa, and a UEL of 11.8%. Furthermore, the BGS Mg-8.2Gd-3.8Y-1.0Zn-0.4Zr alloy reaches a YS of 385 MPa, a UTS of 420 MPa, and a UEL of 19%. Beyond these strength and ductility characteristics, BGS Mg alloys have also been shown to reduce tension–compression asymmetry [51], good superplasticity [52], fatigue performance [53], fracture toughness [54], and damping capacity [55]. The BGS holds significant potential to pave the way for the future development of high-performance Mg alloys.

In polycrystalline metals, macroscopic uniform deformation is commonly known as a result of local coordinated deformation among different-oriented grains, leading to the generation of additional dislocations near grain boundaries [56,57], usually called geometrically necessary dislocations (GNDs) [58]. The long-range stress field is generated by the pileup of GNDs, which is termed back stress [59]. For BGSs, the presence of FG and CG means that coordinated deformation exists not only among different-oriented grains but also among different-sized grains, and the size effect has a higher local coordinated deformation than the orientation one. During the deformation of BGSs, CGs accumulated more strain, causing a larger strain gradient on the CG side of the heterostructure interface, increasing the number of GND pileups, and forming a larger back stress field within the CGs, thus enhancing the strength and strain hardening of the CGs and the overall BGS [60,61,62]. Meanwhile, the presence of back stress in CGs means that there is an equal balancing force at the interface in FGs, forming forward stress within the FGs. Forward stress can promote dislocation dipole annihilation near the interface or dislocation starvation [4,63], causing the local dislocation density to reduce. The unique feature of BGS Mg is that the non-basal slip in CG is more activated during the later stage of deformation than homogeneous Mg. This is because CG captures cracks and shear bands from the FGs, combined with the internal back stress, which causes the local shear stress to reach the critical value for non-basal slip, enhancing the coordinated deformation capability of the CGs and further improving the plasticity of BGSs [64].

Numerous experimental studies have investigated that the HDI effect in BGS Mg is influenced by the grain size [30], volume fraction [15], shape [34,42,46,65,66,67], and basal texture intensity [68,69,70] of CG. However, due to time and preparation costs, a systematic and comprehensive study of the influence of CG configurations is limited. Therefore, computational simulation is used to assist experimental research. Zhu et al. [61] and Li et al. [62] developed a micromechanical model for BGSs using the Mori–Tanaka mean-field scheme and strain gradient theory of plasticity and examined the influence of CG configuration on back stress and overall mechanical properties. Huang et al. [71] and Wang et al. [72] introduced a heterogeneous interface-affected zone between FG and CG regions in BGSs into the material’s plastic constitutive relationship, employing finite element methods to study the impact of CG configuration on the HDI effect. These studies focused on Cu, Ni, and Ti, while for Mg alloys, although some researchers have used the VPSC model to study the influence of texture on the BGS, the HDI effect was not considered [73,74]. There is still a lack of systematic studies on the effects of CG configuration on mechanical responses in BGS Mg.

In this work, using a micromechanics mean-field model combined with the strain gradient theory of plasticity, the effects of CG size, volume fraction, aspect ratio, and texture intensity on the HDI effect are studied in Mg-6.0Zn-0.5Zr (wt.%) (ZK60) alloys, in order to provide guidance on high-performance Mg alloys from a computational design.

## 2. Model Description

### 2.1. Geometric Modeling of the Mean-Field Model for Mg Alloys with Bimodal Grain Size Distribution

In the micromechanics mean-field model, BGS Mg alloys are treated as two-phase composites with a continuous FG matrix and ellipsoidal CG inclusions uniformly embedded within them. In subsequent sections, the following notation is used: Subscripts ‘(0)’, ‘(1)’, and ‘H’ denote variables related to the matrix, inclusion, and overall properties, respectively. The *italic* characters represent scalars, the lower-case bold characters represent second-order tensors, and the upper-case bold characters represent fourth-order tensors.

**Figure 2:**
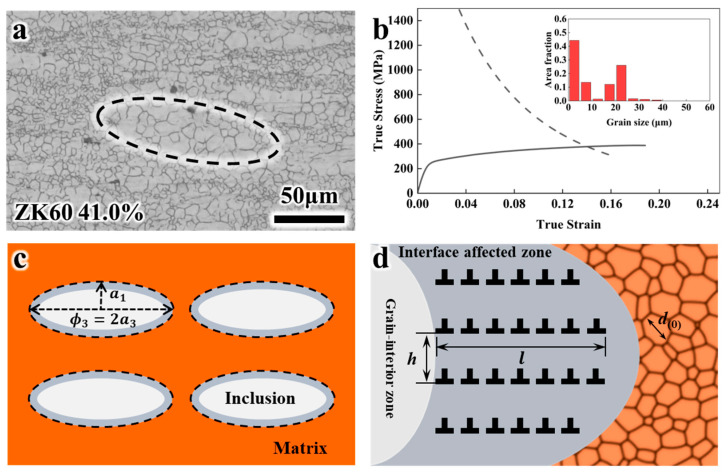
Geometric modeling schematic of bimodal grain size distribution. (**a**) Optical microscopy for BGS ZK60 with CG volume fraction of 41%. (**b**) strain–stress curve of ZK60. The inset is the area fraction distribution of grains with different sizes. (**c**) Schematic of mean-field model based on ellipsoidal inclusion. (**d**) GND pileup at the interface-affected zone (*l*—length of dislocation pileup; *h*—spacing between neighboring pileups in coarse grain (CG); *d*_(0)_—grain size of matrix fine grain (FG); $a_{i}$—axis radius; $\phi_{i}$—axis diameter. Subscripts ‘1’ and ‘2’ represent the minor axes of the ellipsoid, and ‘3’ represents the major axis).

Taking the rolled ZK60 Mg alloy sheet as an example, the microstructure of the BGS in Figure 2a exhibits an FG matrix and CG ellipsoidal inclusions, and the stress–strain curve is shown in Figure 2b, with the inset displaying the grain distribution. This microstructure can be represented as a composite material, as illustrated in Figure 2c. The orange region represents the matrix, the light gray region represents the inclusions, and the dark gray region represents the interface-affected (IA) zone. The IA region is characterized by the accumulation of GNDs in the CG as a result of the local coordinated deformation between the CG and FG. Based on the strain gradient theory [75], the GND pileup length is only a few microns, compared with the CG length in tens of microns.

For the ellipsoidal CG phase, $a_{1}$ and $a_{2}$ denote the semi-minor axes (assuming $a_{1}=a_{2}$), while $a_{3}$ represents the semi-major axis. The aspect ratio is defined as $R=a_{3}/a_{1}$. To account for multiple CGs within the CG phase (Figure 2a), a distinction is made between the shaft diameter ($\phi_{3}$) of the ellipsoidal CG phase and the grain size ($d_{\left(1\right)}$) of CG. The grain size of the FG is denoted as $d_{\left(0\right)}$.

Figure 2d illustrates the dislocation pileups inside the CG. The length of dislocation pileups [75] is given by $l=18\alpha^{2}{\left(\mu /\sigma^{y}\right)}^{2}b$, where *α* is the Taylor coefficient [76,77], *μ* is the shear modulus, $\sigma^{y}$ is the YS, and *b* is the magnitude of the Burgers vector. The spacing between the dislocation pileup arrays is denoted as *h*. Based on this description, the volume fraction of IA region is expressed as follows:

(1) $$f_{IA}=\frac{V_{IA}}{V_{\left(1\right)}}=1-\frac{\left(\phi_{3}-2l\right){\left(\phi_{1}-2l\right)}^{2}}{\phi_{3}{\phi_{1}}^{2}}$$

where *f* is the volume fraction and *V* is the volume. The constitutive behavior of the IA region is incorporated into the CG one in a volume-averaged way [78] to estimate the back stress accurately.

### 2.2. Constitutive Model for Homogeneous Structure Alloys

#### 2.2.1. Dislocation Hardening Model for Homogeneous Structure Phase

Understanding the mechanical behaviors of constituent phases is crucial for determining the overall mechanical response of heterogeneous structures. In homogeneous Mg alloys, dislocations are primarily the statistically stored dislocations (SSDs) [75], which are randomly generated, accumulated, and annihilated within the material, resulting in isotropic strain hardening. The relationship between flow stress ($\sigma^{flow}$) and dislocation density can be described by the Taylor relationship [79].

(2) $$\sigma^{flow}\left(\varepsilon^{p}\right)=\sigma^{y}+\beta \sqrt{\rho \left(\varepsilon^{p}\right)}$$

Here, $\varepsilon^{p}$ is the effective plastic strain, $\sigma^{y}$ is yield strength, β=Mαμb, and *M* is the Taylor factor.

#### 2.2.2. Texture Effect on Homogeneous Structure Phase

In the rolled Mg sheet, strong basal texture is characterized by most grains having a *c*-axis direction within 30° of the normal direction of the plate [80], and the basal texture intensity ($f^{tex}$) is defined as the content of hard-oriented grains. Here, the flow stress in homogeneous structure Mg ($\sigma^{flow}\left(f^{tex}\right)$) is calculated using the VPSC model [81], associated with a different $f^{tex}$, in which each grain assumes a spherical shape. The $f^{tex}$ affects the yield strength and dislocation density evolution of homogeneous structure Mg. The texture effects on yield strength ($\sigma^{y,tex}\left(f^{tex}\right)$) and dislocation density evolution ($\rho^{tex}\left(f^{tex},\varepsilon^{p}\right)$) can be defined as the deviation between the VPSC calculated value and the initial one; the $\sigma^{flow}\left(f^{tex}\right)$ is further expressed as

(3) $$\begin{array}{c} \sigma^{flow}\left(f^{tex},\varepsilon^{p}\right)=\sigma^{y}\left(f^{tex}\right)+\beta \sqrt{\rho \left(f^{tex},\varepsilon^{p}\right)}\\ \;\;\;\;\;\;\;\;\;\;\;\;\;\;\;\;\;\;\;\;\;\;\;\;\;\;\;\;\;\;\;\;\;\;\;\;\;\;\;\;\;\;\;\;\;\;\;\;\;\;\;\;\;\;\;\;\;\;\;\;\;\;\;\;\;\;\;\;\;\;=\sigma^{y0}+\sigma^{y,tex}\left(f^{tex}\right)+\beta \sqrt{\rho^{0}\left(\varepsilon^{p}\right)+\rho^{tex}\left(f^{tex},\varepsilon^{p}\right)} \end{array}$$

where $\sigma^{y0}$ and $\rho^{0}$ are the initial yield strength and the SSD density, respectively, and the $\sigma^{y,tex}$ and $\rho^{tex}$ are the corresponding texture affected terms.

### 2.3. Mori–Tanaka Mean-Field Scheme Framework of Dual-Phase Composite

Based on the Mori–Tanaka scheme [82,83], a micromechanics mean-field model is employed to calculate the overall mechanical response of the dual-phase composite and the strain partitioning between the two phases associated with the secant moduli to handle the plastic behavior of the matrix [84]. In this method, both the inclusions and matrix are assumed to be isotropic solids, and the secant moduli tensor of the matrix ($L_{\left(0\right)}^{s}$) is given by

(4) $$L_{\left(0\right)}^{s}=3\kappa_{\left(0\right)}I^{h}+2\mu_{\left(0\right)}^{s}I^{d}=\frac{E_{\left(0\right)}^{s}}{\left(1-2v_{\left(0\right)}^{s}\right)}I^{h}+\frac{E_{\left(0\right)}^{s}}{\left(1+v_{\left(0\right)}^{s}\right)}I^{d}$$

where $I^{h}$ and $I^{d}$ represent the isotropic operator and the deviatoric operator, respectively. $I_{ijkl}^{h}=\frac{1}{3}\delta_{ij}\delta_{kl}$ and $I_{ijkl}^{d}=\frac{1}{2}\left(\delta_{ik}\delta_{jl}+\delta_{il}\delta_{jk}-\frac{2}{3}\delta_{ij}\delta_{kl}\right)$, where $\delta_{ij}$ is the Kronecker tensor. $\kappa_{\left(0\right)}$ and $\mu_{\left(0\right)}^{s}$ are the bulk modulus and secant shear modulus of the matrix as an isotropic solid. 

Accordingly, $E_{\left(0\right)}^{s}$ and $v_{\left(0\right)}^{s}$ are the secant Young’s modulus and Poison’s ration of the matrix under uniaxial loading, respectively, and the expressions are given by Weng [84] as follows:

(5) $$E_{\left(0\right)}^{s}=\frac{\sigma_{\left(0\right)}^{flow}}{\varepsilon_{\left(0\right)}^{e}+\varepsilon_{\left(0\right)}^{p}}={\left(\frac{1}{E_{0}}+\frac{\varepsilon_{\left(0\right)}^{p}}{\sigma_{\left(0\right)}^{flow}}\right)}^{-1}$$

(6) $$v_{\left(0\right)}^{s}=\frac{1}{2}-\left(\frac{1}{2}-v_{\left(0\right)}\right)\frac{E_{\left(0\right)}^{s}}{E_{\left(0\right)}}$$

Evidently, during the elastic deformation stage, $E_{\left(0\right)}^{s}=E_{0}$ and $v_{\left(0\right)}^{s}=v_{\left(0\right)}$. The isotropic solid assumption is used to simplify the model. Although it may introduce slight quantitative deviations, it does not significantly affect the main conclusions regarding the impact of BGS configuration on the HDI effect and overall response of the BGS under uniaxial tension [85,86,87].

The core idea of the Mori–Tanaka model lies in its consideration of the following:

(i) Using the Eshelby equivalent inclusion method, the heterogeneous inclusions are equivalent to an inclusion with the same properties as the matrix. To not change the stress–strain field of inhomogeneity, a total eigenstrain ($\varepsilon^{*T}$) is introduced to satisfy the following relationship [69]:
  (7) $$\varepsilon^{*T}=\varepsilon^{*}+{\langle \varepsilon^{p}\rangle }_{\left(1\right)}$$
  where $\varepsilon^{*}$ is the equivalent eigenstrain, and ${\langle \varepsilon^{p}\rangle }_{\left(1\right)}$ is the plastic strain of inclusion.
(ii) During plastic deformation, the average stress in inclusions can be calculated by
  (8) $${\langle \sigma \rangle }_{\left(1\right)}=L_{\left(1\right)}\left({\langle \varepsilon \rangle }_{\left(1\right)}-{\langle \varepsilon^{p}\rangle }_{\left(1\right)}\right)=L_{\left(0\right)}^{s}\left({\langle \varepsilon \rangle }_{\left(1\right)}-{\langle \varepsilon^{p}\rangle }_{\left(1\right)}-\varepsilon^{*}\right)$$
  where $L_{\left(1\right)}$ and ${\langle \varepsilon \rangle }_{\left(1\right)}$ are the elastic stiffness tensor and total strain of inclusion, respectively. The difference in deformation characteristics between two phases leads to an inter-phase strain partitioning, which is denoted as a perturbed strain ($\varepsilon^{d}$), i.e., ${\varepsilon^{d}=\langle \varepsilon \rangle }_{\left(1\right)}-{\langle \varepsilon \rangle }_{\left(0\right)}$. $\varepsilon^{d}$ is linearly related to $\varepsilon^{*T}$ through the Eshelby tensor $S_{\left(1\right)}^{s}$, i.e., $\varepsilon^{d}=S_{\left(1\right)}^{s}\varepsilon^{*T}$, and the details of $S_{\left(1\right)}^{s}$ is in Appendix B.
(iii) The strain in the matrix (${\langle \varepsilon \rangle }_{\left(0\right)}$) can be expressed as ${\langle \varepsilon \rangle }_{\left(0\right)}=\varepsilon^{0}+\tilde{\varepsilon }$, where $\varepsilon^{0}$ is the equivalent applied strain given by $\varepsilon^{0}={L_{\left(0\right)}^{s}}^{-1}\sigma^{A}$ with the applied stress of $\sigma^{A}$, and $\tilde{\varepsilon }$ is the interactive strain caused by the neighboring inclusions [82,83]. Thus, Equation (8) can be further expressed by
  (9) $$L_{\left(1\right)}\left(\varepsilon^{0}+\tilde{\varepsilon }+S_{\left(1\right)}^{s}\varepsilon^{*T}-{\langle \varepsilon^{p}\rangle }_{\left(1\right)}\right)=L_{\left(0\right)}^{s}\left[\varepsilon^{0}+\tilde{\varepsilon }+\left(S_{\left(1\right)}^{s}-I\right)\varepsilon^{*T}\right]$$

Based on Weng’s work [84], the average stress $\langle \sigma \rangle$ of each phase under applied stress $\sigma^{A}$ can be expressed as follows:

(10) $${\langle \sigma \rangle }_{\left(0\right)}=B_{\left(0\right)}\sigma_{A}-C_{\left(0\right)}{\langle \varepsilon^{p}\rangle }_{\left(1\right)}$$

(11) $${\langle \sigma \rangle }_{\left(1\right)}=B_{\left(1\right)}\sigma_{A}+C_{\left(1\right)}{\langle \varepsilon^{p}\rangle }_{\left(1\right)}$$

where $B_{\left(0\right)}=I+f_{\left(1\right)}\left(S_{\left(1\right)}^{s}-I\right)Q_{\left(1\right)}\left(L_{\left(1\right)}-L_{\left(0\right)}^{s}\right)$, $C_{\left(0\right)}=f_{\left(1\right)}L_{\left(0\right)}^{s}L_{\left(1\right)}\left(S_{\left(1\right)}^{s}-I\right)Q_{\left(1\right)}$, $B_{\left(1\right)}=I-f_{\left(0\right)}\left(S_{\left(1\right)}^{s}-I\right)Q_{\left(1\right)}\left(L_{\left(1\right)}-L_{\left(0\right)}^{s}\right)$, $C_{\left(1\right)}=f_{\left(0\right)}L_{\left(0\right)}^{s}L_{\left(1\right)}\left(S_{\left(1\right)}^{s}-I\right)Q_{\left(1\right)}$, and $Q_{\left(1\right)}={\left[\left(L_{\left(1\right)}-L_{\left(0\right)}^{s}\right)\left(f_{\left(1\right)}I+f_{\left(0\right)}S_{\left(1\right)}^{s}\right)+L_{\left(0\right)}^{s}\right]}^{-1}$. The average strain of the dual-phase composite can be given by [84]

(12) $$\langle \varepsilon_{H}\rangle =\varepsilon^{0}-f_{\left(1\right)}Q_{\left(1\right)}\left[\left(L_{\left(1\right)}-L_{\left(0\right)}^{s}\right)\varepsilon^{0}-L_{\left(1\right)}{\langle \varepsilon^{p}\rangle }_{\left(1\right)}\right]$$

### 2.4. Local Flow Stress of CG and FG Phases Based on Dislocation Theory

Under the mean-field framework, strain gradient theory is introduced through the perturbed strain term to examine the microstructure characteristics of CG and FG regions, based on a phenomenological revision from the experimental results. Equation (2) is revised to account for these HDI effects, making it applicable for evaluating the strain-hardening behaviors of each phase in BGS Mg alloys.

#### 2.4.1. Back Stress Hardening in CG Phase

During the early stage of deformation, the CG region undergoes plastic deformation, which leads to the formation of the IA zone and then extends toward the CG region. The effective plastic strain gradient can be expressed as [88]

(13) $$\eta_{\left(1\right)}=\frac{\sqrt{3}\left(\varepsilon_{\left(1\right)}^{p}-\varepsilon_{\left(0\right)}^{p}\right)}{\phi_{1}}\approx \frac{\sqrt{3}\varepsilon^{d}}{\phi_{1}}$$

where ∆γ is the effective plastic strain partitioning, and $\varepsilon^{d}=\sqrt{\frac{2}{3}{\langle \varepsilon^{d}\rangle }^{\prime }:{\langle \varepsilon^{d}\rangle }^{\prime }}$ is the effective perturbed strain with the deviatoric strain tensor of $\varepsilon^{\prime }=I^{d}\varepsilon$.

The corresponding GND density ($\rho_{\left(1\right)}^{G})$ and the back stress ($\sigma_{\left(1\right)}^{B}$) within the CG region can be given as [88]

(14) $$\rho_{\left(1\right)}^{G}=\frac{\eta_{\left(1\right)}}{b}\frac{l}{\phi_{3}}=\frac{∆\gamma }{b}\frac{l}{\phi_{1}\phi_{3}}$$

(15) $$\sigma_{\left(1\right)}^{B}=\sqrt{3}f_{IA}A\frac{∆\gamma }{Nb}\frac{\phi_{1}}{l}$$

where l is the GND pileup length, *h* is the mean spacing between pileups, $N=\phi_{1}/h$ is the number of dislocation pileups, and the constant $A=\mu b/\left[\pi \left(1-v\right)\right]$. More details about can be found in [88].

#### 2.4.2. Forward Stress Softening in FG Phase

During larger deformations, the high-level stress concentration from the GND pileup in the CG/FG interface leads to the FG dislocation escape from the interface [4,89,90,91,92] and brings about a reduction in FG dislocation density ($\rho_{\left(0\right)}^{I}$), resulting in local FG softening. The reduction in dislocation density ($\rho_{\left(0\right)}^{I}$) can be calculated using the following equation [90,93]:

(16) $$\rho_{\left(0\right)}^{I}=\lambda \frac{{\sigma_{\left(1\right)}^{B}}^{2}}{A\langle \Lambda \rangle }=\frac{\sigma_{\left(0\right)}^{F}}{A\langle \Lambda \rangle }$$

where $\langle \Lambda \rangle$ is the mean free path of dislocation dipole annihilation and $\lambda =\lambda_{0}f_{\left(0\right)}/f_{\left(1\right)}$ is the proportion coefficient. The detailed derivation can be found in [90,93]. Therefore, forward stress is expressed as

(17) $$\sigma_{\left(0\right)}^{F}=\lambda {\sigma_{\left(1\right)}^{B}}^{2}l/A$$

as a proportion of the stress of the pileup tip near the interface.

#### 2.4.3. Forward and Back Stress Compromised by Microcracks in CG Phase

During severe plastic deformation, microcracks nucleate derived from pre-existed pileup dislocations and propagate within the FG and CG phases [94,95]. To release the stress field at the crack tip, dislocations form around the crack tip [96] and stop near the GBs, generating an additional back stress effect $\sigma_{\left(1\right)}^{C}$, which can be expressed as [61]

(18) $$\sigma_{\left(1\right)}^{C}=\frac{\sqrt{3}A}{d_{\left(1\right)}}N^{C}=\frac{\sqrt{3}A}{d_{\left(1\right)}}N_{0}\left[1-exp\left(-\frac{h_{c}\varepsilon_{\left(1\right)}^{p}}{bN_{0}}\right)\right]$$

where $N^{C}=N_{0}\left\{1-\exp\left[-h_{c}\varepsilon_{\left(1\right)}^{p}/\left(bN_{0}\right)\right]\right\}$ represents the number of dislocations stopped at the GBs, *h*_c_ is the mean spacing between forthcoming pileups induced by cracks, and $N_{0}$ is the maximum number of dislocations to match *h*_c_.

#### 2.4.4. Effect of Basal Texture on Flow Stress in BGS Mg

In BGS Mg alloys, the effect of basal texture on flow stress is critical. Since the DRXed FG phase is made by random-oriented grains, the effects of FG on mechanical properties are primarily from the average grain size, while the plastic deformation behaviors of the un-DRXed CG phase is greatly affected by its basal texture characteristics. 

To investigate this influence, a random-oriented polycrystalline material ($f^{tex}=0$) is used as the initial configuration of the CG phase [97] with the flow stress of ($\sigma_{\left(1\right)}^{0}$). The $f^{tex}$ increases in terms of replacing the initial orientation of grains with hard orientation. Referring to Equation (3), the flow stress of the CG phase $\sigma_{\left(1\right)}^{flow}\left(f^{tex},\varepsilon_{\left(1\right)}^{p}\right)$ considering the texture effect is expressed as follow

(19) $$\begin{array}{c} \sigma_{\left(1\right)}^{flow}\left(f^{tex},\varepsilon_{\left(1\right)}^{p}\right)=\sigma_{\left(1\right)}^{y}\left(f^{tex}\right)+\beta \sqrt{\rho_{\left(1\right)}\left(f^{tex},\varepsilon_{\left(1\right)}^{p}\right)}\\ \;\;\;\;\;\;\;\;\;\;\;\;\;\;\;\;\;\;\;\;\;\;\;\;\;\;\;\;\;\;\;\;\;\;\;\;\;\;\;\;\;\;\;\;\;\;\;\;\;\;\;\;\;\;\;\;\;\;\;\;\;\;=\sigma_{\left(1\right)}^{y0}+\sigma_{\left(1\right)}^{y,tex}\left(f^{tex}\right)+\beta \sqrt{\rho_{\left(1\right)}^{0}\left(\varepsilon^{p}\right)+\rho_{\left(1\right)}^{tex}\left(f^{tex},\varepsilon_{\left(1\right)}^{p}\right)} \end{array}$$

The $\sigma_{\left(1\right)}^{flow}$ with different $f^{tex}$ configurations can be obtained from the VPSC model as a revised constitutive input of CG. The detailed calculation process is provided in Appendix A. 

#### 2.4.5. Flow Stress of Each Phase and HDI Effect in BGS Mg

The HDI effect is divided into FG phase softening from forward stress and CG phase hardening from back stress. Back stress hardening can be further divided into inter-phase back stress and crack back stress. The corresponding revised flow stress of the CG and FG phases can be written in the following form:

(20) $$\sigma_{\left(0\right)}^{rev}=\sigma_{\left(0\right)}^{y0}+\beta \sqrt{\rho_{\left(0\right)}^{0}-\rho_{\left(0\right)}^{I}}$$

(21) $$\sigma_{\left(1\right)}^{rev}=\sigma_{\left(1\right)}^{y0}+\sigma_{\left(1\right)}^{y,tex}+\beta \sqrt{\rho_{\left(1\right)}^{0}+\rho_{\left(1\right)}^{tex}+\rho_{\left(1\right)}^{G}}+\sigma_{\left(1\right)}^{B}+\sigma_{\left(1\right)}^{C}$$

The calculation formulas for CG phase hardening, FG phase softening, and the HDI effect are as follows:

(22) $$\sigma^{BS}=\sigma_{\left(1\right)}^{rev}-\beta \sqrt{\rho_{\left(1\right)}^{0}+\rho_{\left(1\right)}^{tex}}-\sigma_{\left(1\right)}^{y0}-\sigma_{\left(1\right)}^{y,tex}$$

(23) $$\sigma^{FS}=\sigma_{\left(0\right)}^{rev}-\beta \sqrt{\rho_{\left(0\right)}^{0}}-\sigma_{\left(0\right)}^{y0}$$

(24) $$\sigma^{HDI}=f_{\left(1\right)}\sigma^{BS}+f_{\left(0\right)}\sigma^{FS}$$

### 2.5. Numerical Implementation

Finally, it is necessary to explain the connection between the Mori–Tanaka scheme and the constitutive relationships of each phase constructed in this section. This relationship is constructed through the *J*_2_ deformation theory. According to the yield criterion of the *J*_2_ deformation theory, the flow stress from Equations (20) and (21) is equal to the von Mises equivalent stress of each phase from Equations (10) and (11):

(25) $$\sigma_{\left(i\right)}^{rev}=\sigma_{\left(i\right)}^{eq}=\sqrt{\frac{3}{2}{\langle \sigma \rangle }_{\left(i\right)}^{\prime }:{\langle \sigma \rangle }_{\left(i\right)}^{\prime }}$$

where ${\langle \sigma \rangle }_{\left(i\right)}^{\prime }=I^{d}{\langle \sigma \rangle }_{\left(i\right)}$ is the deviatoric stress tensor of the matrix (0) or inclusion (1) phases from Equations (10) and (11). And the plastic strain of each phase can be given as

(26) $${\langle \varepsilon^{p}\rangle }_{\left(i\right)}=\frac{3}{2}\frac{\varepsilon_{\left(i\right)}^{p}}{\sigma_{\left(i\right)}^{rev}}{\langle \sigma \rangle }_{\left(i\right)}^{\prime }$$

The UTS and UEL is evaluated by the Considère criterion [77]:

(27) $$\theta_{H}=\frac{\partial \sigma_{11}^{A}}{\partial \varepsilon_{H}}<\sigma_{11}^{A}$$

where $\theta_{H}$ is the strain-hardening rate of the composite, and $\varepsilon_{H}=\sqrt{\frac{2}{3}{\langle \varepsilon \rangle }_{H}^{\prime }:{\langle \varepsilon \rangle }_{H}^{\prime }}$ is the von Mises equivalent strain from Equation (12).

The deformation of the composite, under a given applied stress increment, ${\sigma_{11}^{A}}_{n+1}=({\sigma_{11}^{A}}_{n}+∆{\sigma_{11}^{A}}_{n})$, can be determined by solving the system of equations formed by combining Equations (10)–(12), (20), (21), (25), and (26). This allows for the calculation of ${\langle \varepsilon^{p}\rangle }_{\left(0\right)}$, ${\langle \varepsilon^{p}\rangle }_{\left(1\right)}$, and $\langle \varepsilon_{H}\rangle$. This process subsequently allows for the calculation of stress and strain for each phase and the whole composite, respectively. The plastic deformation of the BGS can be specifically divided into four stages:

(a) Soft-CG phase yielding and hard-FG phase elastic deformation: $\varepsilon_{\left(i\right)}^{p}=0$, $\sigma_{\left(1\right)}^{eq}=\sigma_{\left(1\right)}^{Y}={\sigma_{11}^{A}}_{n+1}$, and $L_{\left(0\right)}^{s}=L_{\left(0\right)}$;
(b) Hard-FG phase yielding and soft-FG CG phase plastic deformation: $\varepsilon_{\left(0\right)}^{p}=0$, $\sigma_{\left(0\right)}^{eq}=\sigma_{\left(0\right)}^{Y}$, $\sigma_{\left(1\right)}^{rev}=\sigma_{\left(1\right)}^{eq}$, and $L_{\left(0\right)}^{s}=L_{\left(0\right)}$;
(c) Plastic deformation occurs in both CG and FG phases before necking: $\sigma_{\left(i\right)}^{rev}=\sigma_{\left(i\right)}^{eq}$;
(d) Onset of necking: When the relationship between the strain-hardening rate of the compound and stress satisfies Equation (27), the calculation is complete, yielding the UTS and UEL.

## 3. Results and Discussion

The material parameters of ZK60 are derived from research [95]. $\langle \Lambda \rangle$ is set to 50 nm based on the length of dislocation arrays observed in Mg alloys in the experiment report [98]. Other parameters are obtained by fitting the mechanical response of experimental samples at different deformation stages according to the method in reference [77]. The values are summarized in Table 1.

### 3.1. Comparison with Experiments on Yield Strength and Strain Hardening

**Figure 3:**
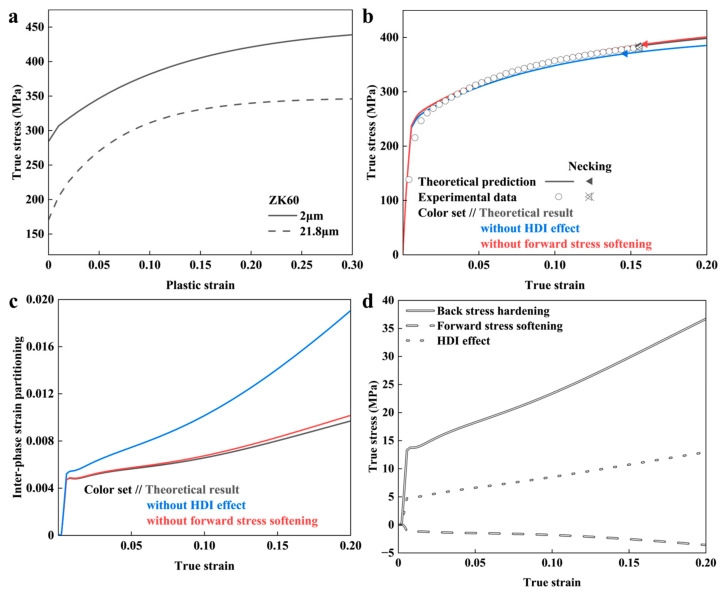
The predicted results from the mean-field model for BGS ZK60 during tensile loading. (**a**) Flow stress of the individual constituent phases [100]. (**b**) Predicted overall stress–strain response. Results for cases with and without considering extra strengthening are included. (**c**) Inter-phase strain partitioning. (**d**) HDI effect.

In order to verify the validation of the proposed model, the mechanical properties of BGS ZK60 were predicted based on its geometric parameters as described in the previous work [99]. The volume fraction *f*_(1)_, average grain size *d*_(1)_, major axis diameter *ϕ*_1_, and axis ratio *R* in the CG phase are set to 41%, 23 μm, 130 μm, and 3, respectively. The average grain size of the FG phase is 2 μm. The $\sigma_{\left(i\right)}^{0}$ for the FG and CG phases of ZK60 are shown in Figure 3a. The FG $\sigma_{\left(0\right)}^{0}$ is taken from [100]. The CG $\sigma_{\left(1\right)}^{0}$ is taken from our group’s experimental results, and $f^{tex}$ is set to 0. The $\sigma^{y0}$ of FG and CG is 284 MPa and 170 MPa, respectively.

The model prediction results are shown in Figure 3b. The predicted YS, UTS, and UEL of BGS ZK60 are 261 MPa, 384 MPa, and 15.6%, respectively. These values agree well with the experimental values of 258 MPa, 381 MPa, and 15.6%.

Removing the forward stress softening results in the UEL of the BGS changing from 15.6% to 16.0%. It is indicated that within the HDI effect, forward stress softening slightly reduces the strain-hardening ability of the BGS. As shown in Figure 3c, forward stress reduces the inter-phase strain partitioning, thereby weakening the HDI effect. This is because dislocation dipole annihilation reduces the strength of the FG phase near the heterogeneous interface.

Removing the HDI effect reduces the UEL of the BGS from 15.6% to 14.7%, indicating a positive impact of the HDI effect on the mechanical properties of the BGS ZK60.

Figure 3d illustrates the relationship between the HDI effect and applied strain on BGS ZK60. At an applied strain of 15.6%, the back stress, forward stress, and HDI effect in ZK60 are 31 MPa, −3 MPa, and 11 MPa, respectively. It should be noted that the intragranular back stresses arising from the heterogeneous dislocation distribution such as dislocation cells and walls [59] are already included in the $\sigma_{\left(i\right)}^{ref}$.

**Figure 4:**
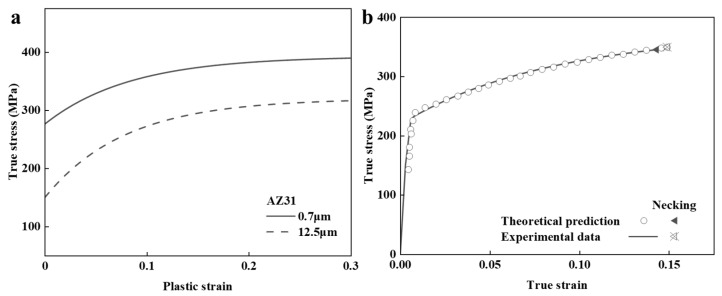
The predicted results from the mean-field model for BGS AZ31 with average grain sizes of the FG and CG phases of 0.7 μm and 12.5 μm, respectively, during tensile loading. (**a**) The flow stress of the individual constituent phases from the reference of [47] and (**b**) the predicted overall stress–strain response, in comparison with experimental results [47].

Moreover, the present model can evaluate the mechanical behaviors of BGSs with the nano-sized FG. Taking BGS AZ31 as an example, the average grain size of the FG and CG phases are 0.7 μm and 12.5 μm, respectively [47], with a spherical CG volume fraction of 40%. $\sigma_{\left(i\right)}^{ref}$ was taken from [47], as shown in Figure 4a. *h* and $N_{0}$ were set as 300 nm and 30, respectively. All other parameters were kept consistent with the previous BGS ZK60 model. As shown in Figure 4b, the model successfully predicts the mechanical properties of the BGS AZ31 alloy, after comparing it with the experimental results.

### 3.2. The Influence of Aspect Ratios of Coarse Grains on the Mechanical Response of BGS Mg Alloys

To reveal the influence of the CG aspect ratio (*R*) on the HDI effect and overall properties of the BGS, the mechanical response of BGS ZK60 was calculated for an *R* of 1, 3, 5, 7, 10, 15, and 20. Here, $\phi_{1}$ remains constant, and $\phi_{3}=R\phi_{1}$. All other geometric parameters are kept the same as the verification model.

**Figure 5:**
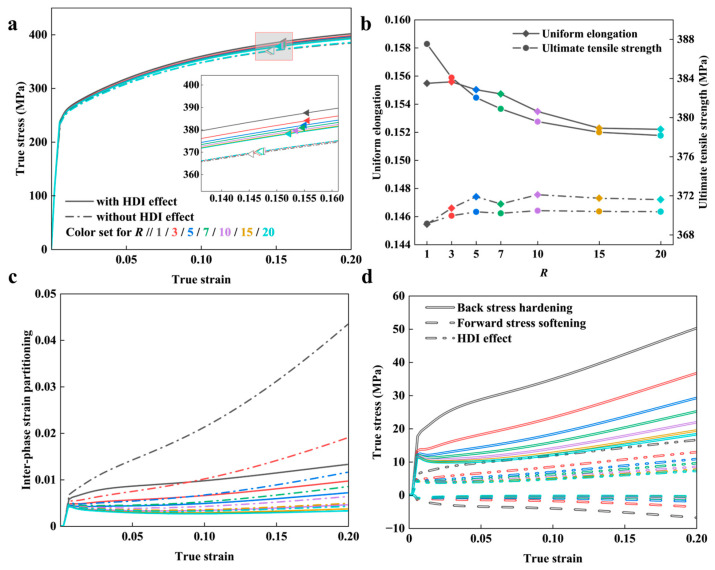
The predicted results from the mean-field model for BGS ZK60 with different aspect ratios, *R*, during tensile loading. (**a**) Predicted overall stress–strain response. Results for cases with and without the HDI effect are included. (**b**) Mechanical performance indexes. (**c**) Inter-phase strain partitioning. (**d**) The HDI effect. Color settings in all subfigures are the same as in subfigure (**a**).

Figure 5a,b show that the *R* has virtually no effect on the YS. When the HDI effect is not considered, increasing the *R* slightly enhances the strain-hardening ability of the BGS Mg alloys. This is evidenced by the UTS increasing from 369 MPa to 370 MPa and the UEL increasing from 14.5% to 14.8%. The improvements in UTS and UEL peak at *R* = 10, and then both decline slightly as *R* increases.

When considering the HDI effect, the strain-hardening ability of BGS ZK60 decreases with increasing *R*, also reaching a critical point around *R* = 10, after which the rate of decrease slows down. Specifically, the UTS decreases from 388MPa to 378MPa and the UEL decreases from 15.6% to 15.2%.

Increasing *R*, as shown in Figure 5c,d, reduces inter-phase strain partitioning, thereby diminishing back stress hardening within the CG phase. And as indicated in Section 3.1, the influence of forward stress is small compared to back stress in the current configuration. This has led to a decrease in the HDI effect, deteriorating the overall performance of the BGS.

### 3.3. Influence of Coarse Grain Size on Mechanical Response of BGS Mg Alloys

To reveal the influence of CG size on the HDI effect and overall properties of BGS ZK60, the mechanical response of BGS ZK60 was calculated at CG sizes of 21.8 μm and 68 μm.

**Figure 6:**
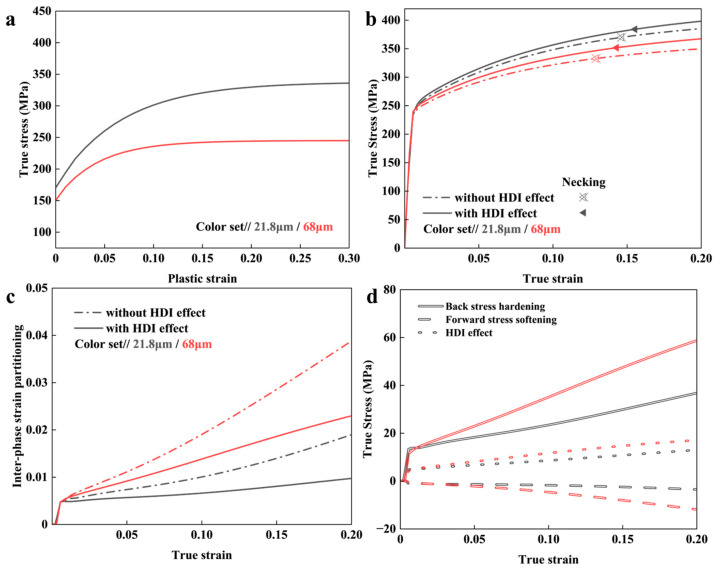
The predicted results from the mean-field model for BGS ZK60 with different CG sizes during tensile loading. (**a**) The flow stress of the individual constituent phases [101] and (**b**) the predicted overall stress–strain response. Results for cases with and without the HDI effect are included. (**c**) Strain partitioning and (**d**) the HDI effect.

Figure 6a presents the $\sigma_{\left(1\right)}^{ref}$ for different CG sizes (21.8 μm and 68 μm [101]). The corresponding theoretical results of BGS ZK60 Mg are shown in Figure 6b. As the CG size increases, the YS of the BGS decreases from 261 MPa to 236 MPa, and the UTS and UEL decrease from 384 MPa to 352 MPa and from 15.6% to 14.3%, respectively.

After eliminating the HDI effect, the UELs of the two samples decreased by 0.9% and 1.4%, respectively, indicating that larger CG sizes lead to a greater enhancement of overall mechanical properties by the HDI effect. As shown in Figure 6c,d, this is because the inter-phase strain partitioning and HDI effect increase with CG size. However, the HDI effect cannot compensate for the strength reduction in the BGS caused by increased CG size.

To evaluate the detailed relationship between the HDI effect and grain size, the back stress was calculated for different grain sizes using Equation (15), assuming a ∆γ of 1%. Using data from [102], the Hall–Petch relationship between YS and grain size was obtained as $\sigma_{y}=168+171d^{-1/2}$. Assuming ideal plasticity for CG (θ=0), the lower bound of CG strength was calculated.

**Figure 7:**
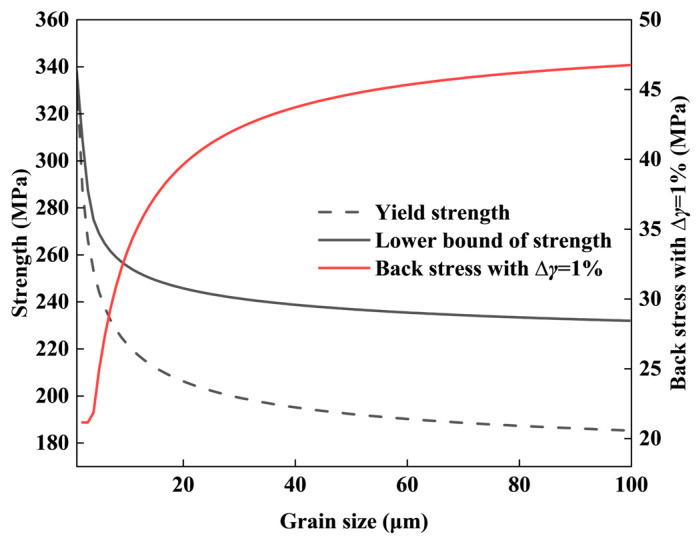
The yield strength, back stress, and lower bound of strength as a function of CG size.

Figure 7 shows that back stress increases with CG size, with the rate of increase slowing around 20~30 μm. Conversely, the lower bound of CG strength decreases with increasing CG size. This indicates that while increasing CG size enhances back stress hardening, it negatively impacts CG strength.

### 3.4. Influence of Volume Fraction of Coarse Grains on Mechanical Response of BGS Mg Alloys

To reveal the influence of the CG volume fraction (*f*_(1)_) on the HDI effect and overall properties, the mechanical response of BGS ZK60 was calculated at an *f*_(1)_ of 5~45%.

**Figure 8:**
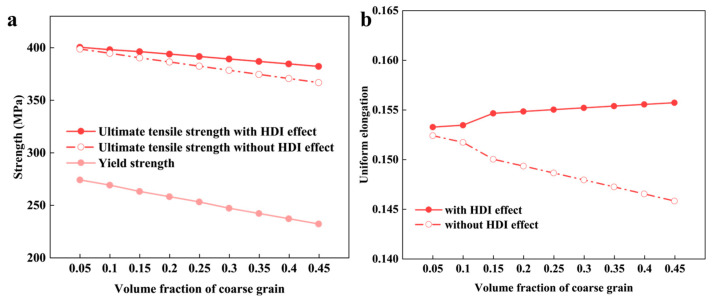
The predicted results from the mean-field model for BGS ZK60 as a function of coarse grain (CG) volume fraction during tensile loading. (**a**) Yield and ultimate tensile strength and (**b**) uniform elongation.

Figure 8 illustrates the effect of CG volume fraction on the YS, UTS, and UEL of BGS ZK60. The results show that as CG volume fraction increases, the YS and UTS of the BGS decrease from 274 MPa to 227 MPa and from 400 MPa to 382 MPa, respectively. The UEL of the BGS initially increased from 15.3% to 15.6%.

Without the HDI effect, the UTS and UEL of the BGS continuously decrease from 399 MPa to 367 MPa and from 15.2% to 14.6% as the CG volume fraction increases, respectively. This suggests that in BGS Mg alloys, the HDI effect effectively mitigates CG’s negative impact on strength and plasticity.

### 3.5. Influence of Texture Intensity of Coarse Grains on Mechanical Response of BGS Mg Alloys

To reveal the influence of CG texture intensity on HDI effects and overall mechanical performance, $\sigma_{\left(1\right)}^{flow}\left(f^{tex}\right)$ with an $f^{tex}$ from 0 to 100% was used to evaluate the mechanical properties of BGS ZK60 sheets.

**Figure 9:**
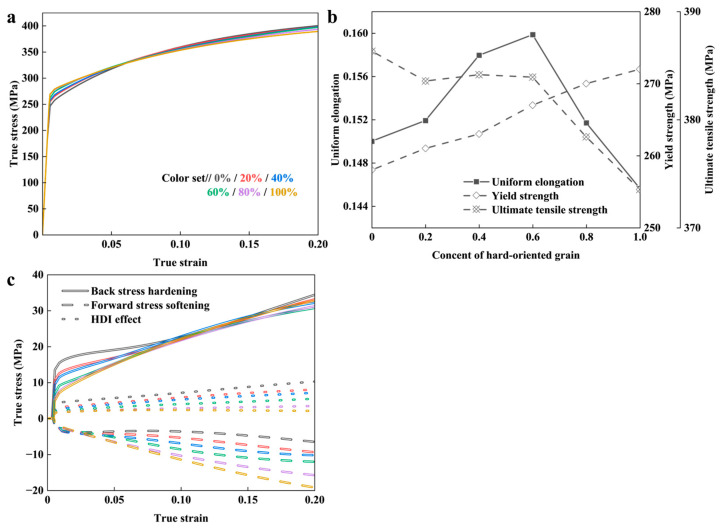
The predicted results from the mean-field model for BGS ZK60 as a function of basal texture intensity under uniaxial tension along the RD direction. (**a**) Predicted overall stress–strain response. (**b**) Yield strength, ultimate tensile strength, and uniform elongation. (**c**) The HDI effect. Same as the color setting of subfigure (**a**).

Theoretical calculation results are shown in Figure 9a. The obtained mechanical performance indexes are shown in Figure 9b. This leads to an increase in the YS of the BGS from 258 MPa to 272 MPa. The UTS of the BGS decreases gradually with an increase in hard-oriented grain content, from 386 MPa to 374 MPa. The UEL of the BGS reaches peak values of 16.0% when the $f^{tex}$ is around 60%.

Figure 9c illustrates that increasing the $f^{tex}$ enhances back stress hardening in the later stages of deformation. However, the HDI effect does not always compensate for the deterioration of strain-hardening capacity in the CG phase caused by increased texture intensity. This results in a continuous decline in the UTS of the BGS, and a peak in UEL.

**Figure 10:**
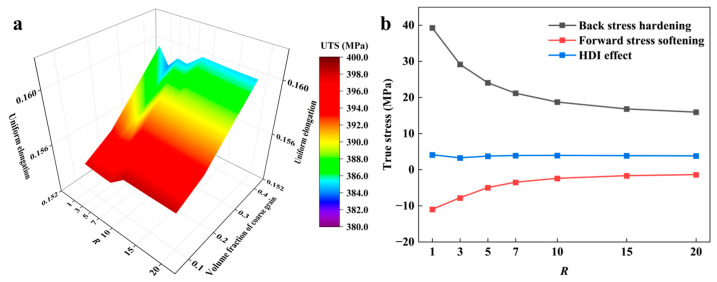
(**a**) The uniform elongation–ultimate tensile strength map with different volume fractions of coarse grain (CG) and *R* under uniaxial tension; (**b**) the HDI effect as a function of *R* with a CG volume fraction of 30% when the applied strain is UEL.

Finally, using the flow stress of 60% hard-oriented grains as a reference flow stress for the CG phase, the combined effect of CG volume fraction and *R* on the mechanical properties of BGS ZK60 was investigated. The results are shown in Figure 10. 

The influence of *R* variation on mechanical properties differs slightly from the situation in Section 3.2. As the *R* increases, both UTS and UEL exhibit a trend of first decreasing and then increasing. However, the final values did not exceed the UTS and UEL at *R* = 1. The reason for this trend is that the strain-hardening capability of CG with $f^{tex}=60\%$ is relatively poor, making it more sensitive to changes in the HDI effect. As shown in Figure 10b, initially, as the *R* increases, the inter-phase strain partitioning decreases, leading to a reduction in the HDI effect, which causes the mechanical properties of CG and the BGS to decrease. However, as the *R* continues to increase, the HDI effect slightly improves, resulting in an enhancement of the mechanical properties of CG and the BGS.

The influence of volume fraction variation on mechanical properties is consistent with Section 3.4, i.e., as the CG volume fraction increases, UTS decreases but UEL increases. When the CG content is 30%, the BGS has a good combination of strength and plasticity.

Based on the above discussion results, a design scheme for BGS Mg alloys can be obtained. In this design, the FG phase serves as the matrix, while the CG phase acts as the inclusion phase. The CG size is around 20~30 μm and the volume fraction is around 30%. *R* is around 1 for ensuring the HDI effect. And CG has a $\left\{0001\right\}\langle 10\bar{1}0\rangle$ basal texture with medium intensity (60% content of hard-oriented grains). This combination enhances the overall strength and plasticity of BGS Mg alloys through the HDI effect.

## 4. Conclusions

In this work, taking BGS ZK60 alloys as an example, the influences of CG size, volume fraction, and texture intensity on the HDI effect and mechanical properties are investigated, using a micromechanics mean-field model, combined with the strain gradient theory of plasticity. The main conclusions are summarized as follows:

(1) The overall mechanical properties decrease with an increase in CG size because the limited HDI effect cannot compensate for the strength and plasticity decrease derived by larger CGs.
(2) An increase in the aspect ratio *R* of CG reduces inter-phase strain partitioning, weakening the HDI effect, which consequently decreases the overall mechanical properties.
(3) With an increase in CG volume fraction, both yield and tensile strength decrease. However, under the influence of the HDI effect, the plasticity of BGS ZK60 increases. When the CG volume fraction is about 30%, there is a good synergistic effect between strength and plasticity.
(4) Enhancing the basal texture intensity improves the BGS ZK60 yield strength and uniform elongation, and they reach the maximum value at the medium texture intensity with 60% hard-oriented CG content. Further increasing the hard-oriented CG content will decrease strength and plasticity instead.

## Figures and Tables

**Table 1:** Descriptions, symbols, and magnitudes of different material parameters of the model.

Type of Parameters	Symbol	Unit	Parameter Value	Reference
Elastic modulus	*E*	GPa	44.4	[95]
Shear modulus	*G*	GPa	17.5	
Poisson’s ratio	*v*		0.27	
Magnitude of Burgers vector	*b*	nm	0.25	
Taylor factor	*M*		3.06	
Taylor constant	*α*		0.30	
Mean free path of dislocation dipole annihilation	$\langle \Lambda \rangle$	nm	50	[98]
Mean spacing between pileups	*h*	nm	2000	
Attenuation coefficient	$\lambda_{0}$		0.1	
Maximum number of dislocations	$N_{0}$		200	
Mean spacing between pileups induced by cracks	$h_{c}$	nm	100	

**Figure A1:**
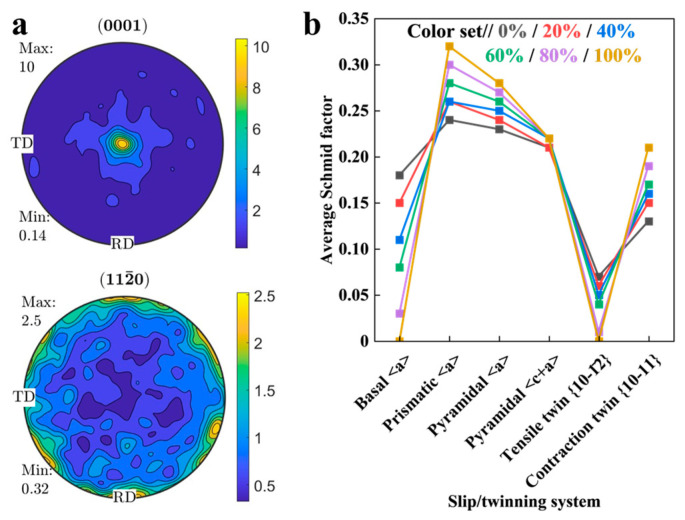
The predicted results from the VPSC model: (**a**) pole figure and (**b**) average Schmid factor for the ZK60 sheet with different contents of hard-oriented grains.

**Figure A2:**
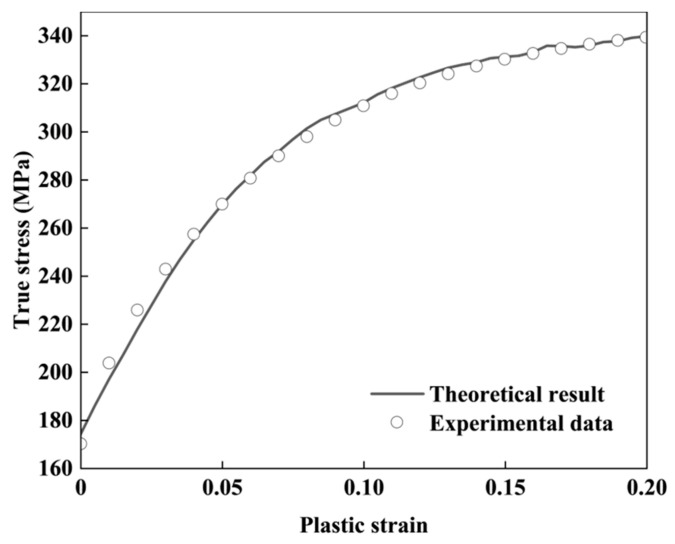
The predicted overall stress–strain response and experimental data under uniaxial tension along the RD direction for the ZK60 sheet with a 21.8 μm average grain size.

**Figure A3:**
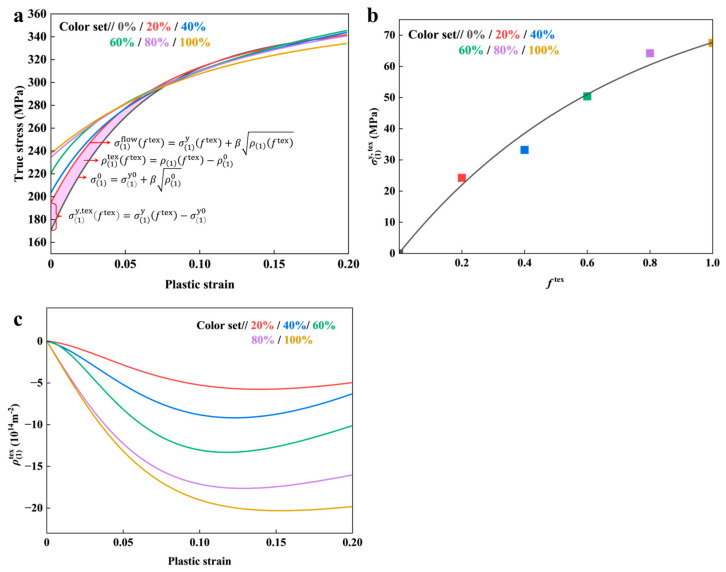
The predicted results under uniaxial tension along the RD direction for the ZK60 sheet with different $f^{tex}$. (**a**) Predicted overall stress–strain response. The texture intensity was affected in terms of (**b**) yield strength and (**c**) dislocation density evloution.

## Data Availability

The research data were generated by the direct application of the model and of its associated equations.

## Appendix A. Calculating the Influence of Texture on Flow Stress Using the VPSC Model

### Appendix A1. Model Description

The whole framework of VPSC can be found in [103,104]. We only briefly describe the crystal plasticity-based strain-hardening relationship at the grain level used in the VPSC model. The visco-plastic constitutive behavior of grains can be expressed by the non-linear rate-sensitivity equation, as follows:

(A1) $${\dot{\varepsilon }}_{ij}^{g}={\dot{\gamma }}_{0}\sum_{s}m_{ij}^{s}{\left(\frac{m_{kl}^{s}\sigma_{kl}^{\prime }}{\tau^{s}}\right)}^{n}$$

where ${\dot{\varepsilon }}_{ij}^{g}$ is the strain rate of the grain, ${\dot{\gamma }}_{0}$ is a reference rate, $\tau^{s}$ is the threshold stress, *n* is the reciprocal of the rate sensitivity, and $\sigma_{kl}^{\prime }$ is the applied deviatoric stress; $m_{ij}^{s}=\frac{1}{2}\left(n_{i}^{s}b_{j}^{s}+n_{j}^{s}b_{i}^{s}\right)$ is the symmetric Schmid tensor associated with the slip/twinning system (s), where $n_{i}^{s}$ and $b_{i}^{s}$ are the normal and Burgers vector of such a slip/twinning system.

The $\tau^{s}$ of each slip/twinning system is calculated using the Voce hardening equation [103,104], which describes its evolution with accumulated shear strain in each grain, and is given by

(A2) $$\tau^{s}=\tau_{0}^{s}+\left(\tau_{0}^{s}+\theta_{1}^{s}\Gamma \right)\left[1-\exp\left(-\Gamma \left|\frac{\theta_{0}^{s}}{\tau_{1}^{s}}\right|\right)\right]$$

where s represents the slip/twin system; $\Gamma =\sum ∆\gamma^{s}$ is the accumulated shear in the grain; $\tau_{0}$, $\tau_{1}$, $\theta_{0}$, and $\theta_{1}$ are initial critical resolved shear stress, back-extrapolated critical resolved shear stress, initial hardening rate, and asymptotic hardening rate, respectively.

To eliminate the influence of other textures, we used a hypothetical polycrystalline aggregate consisting of 1000 spherical grains with randomly generated discrete orientations without texture [97] as the initial reference configuration, allowing us to enhance the basal texture intensity by substituting some of the grains with hard orientation grains which have Bunge orientation angles of (0~30°, 0, 0) and to evaluate the effect of texture on flow stress. The corresponding pole figure of the initial polycrystalline aggregate is shown in Figure A1a. The basal texture intensity ($f^{tex}$) is defined as the content of hard-oriented grains in the polycrystalline aggregate. The $f^{tex}$ of the initial polycrystalline aggregate is zero. The effect of $f^{tex}$ on the activation of these slip/twin systems can be characterized by the average Schmid factor, as follows:

(A3) $${\bar{m}}^{s}=\sum_{g}\frac{f^{g}m_{ij}^{s}\sigma_{ij}}{\sqrt{\sigma_{ij}\sigma_{ij}}}$$

where $f^{g}$ is the volume fraction of grain. This VPSC model considers four types of slip systems and two types of twin systems. The average Schmid factor with different $f^{tex}$ are shown in Figure A1b.

The flow stress of the polycrystalline aggregate with different $f^{tex}$ ($\sigma^{flow}\left(f^{tex},\varepsilon^{p}\right)$) is directly calculated using VPSC. The mechanical properties of the initial polycrystalline aggregate with $f^{tex}=0$, obtained from tensile testing, serve as a reference. The effect of texture intensity on its yield strength ($\sigma^{y,tex}\left(f^{tex}\right)$) and dislocation density evolution ($\rho^{tex}\left(f^{tex},\varepsilon^{p}\right)$) is defined as the deviation between the VPSC calculated values ($\sigma^{y}\left(f^{tex}\right)$ and $\rho \left(f^{tex},\varepsilon^{p}\right)$) and the initial ones ($\sigma^{y0}$ and $\rho^{0}\left(\varepsilon^{p}\right)$). Thus, the influence of $f^{tex}$ on the reference flow stress is further expressed as

(A4) $$\begin{array}{c} \sigma^{flow}\left(f^{tex},\varepsilon^{p}\right)=\sigma^{y}\left(f^{tex}\right)+\beta \sqrt{\rho \left(f^{tex},\varepsilon^{p}\right)}\\ \;\;\;\;\;\;\;\;\;\;\;\;\;\;\;\;\;\;\;\;\;\;\;\;\;\;\;\;\;\;\;\;\;\;\;\;\;\;\;\;\;\;\;\;\;\;\;\;\;\;\;\;\;\;\;\;\;\;\;\;\;\;\;\;\;\;\;\;\;\;\;=\sigma^{y0}+\sigma^{y,tex}\left(f^{tex}\right)+\beta \sqrt{\rho^{0}\left(\varepsilon^{p}\right)+\rho^{tex}\left(f^{tex},\varepsilon^{p}\right)} \end{array}$$

### Appendix A2. Numerical Results

#### Appendix A2.1. Model Validation

The hardening parameters listed in Table A1 are based on the values from [98] and adjusted according to the flow stress of the CG phase obtained from the tensile test, which serves as the flow stress of the initial polycrystalline aggregate. As shown in Figure A2, the predicted results agree well with the tensile data of the CG phase.

**Table A1:** Simulated VPSC hardening parameters of ZK60 alloy under uniaxial tension conditions.

Deformation Mode	Crystallographic Type	$\tau_{0}$ /MPa	$\tau_{1}$ /MPa	$\theta_{0}$ /MPa	$\theta_{1}$ /MPa
Basal <a>	$\left\{0001\right\}\langle 11\bar{2}0\rangle$	8	55	350	120
Prismatic <a>	$\left\{10\bar{1}0\right\}\langle 11\bar{2}0\rangle$	150	50	280	0
Pyramidal <a>	$\left\{10\bar{1}1\right\}\langle 11\bar{2}0\rangle$	300	150	800	0
Pyramidal <c + a>	$\left\{11\bar{2}2\right\}\langle 11\bar{2}3\rangle$	180	300	4200	0
Tension twin	$\left\{10\bar{1}2\right\}\langle 10\bar{1}1\rangle$	80	0	0	0
Contraction twin	$\left\{10\bar{1}1\right\}\langle 10\bar{1}2\rangle$	220	0	0	100

$\tau_{0}$, $\tau_{1}$, $\theta_{0}$, and $\theta_{1}$ are initial critical resolved shear stress, back-extrapolated critical resolved shear stress, initial hardening rate, and asymptotic hardening rate, respectively.

#### Appendix A2.2. The Effect of Basal Texture Intensity on CG Flow Stress

The theoretical results of stress–strain curves for the CG phase with different $f^{tex}$ are shown in Figure A3a. Taking the flow stress of the initial polycrystalline aggregate ($f^{tex}=0$) as a reference, the values of $\sigma_{\left(1\right)}^{y,tex}\left(f^{tex}\right)$ and $\rho_{\left(1\right)}^{tex}\left(f^{tex},\varepsilon^{p}\right)$, derived from Figure A3a, are shown in Figure A3b,c. As $f^{tex}$ increases, the YS of the CG phase increases from 172 MPa to 239 MPa, but the strain-hardening ability gradually weakens, manifesting as a decrease in accumulated dislocation density under the same plastic strain.

## Appendix B. Eshelby Tensor for Isotropic Materials

Based on the geometric model description in the manuscript, the expression for the Eshelby tensor of an ellipsoidal inclusion is as follows:

(A5) $$\begin{array}{c} s_{1111}=s_{2222}=-\frac{3R^{2}}{8\left(1-v\right)\left(1-R^{2}\right)}+\frac{1}{4\left(1-v\right)}\left[1-2v+\frac{9}{4\left(1-R^{2}\right)}\right]g\\ s_{3333}=\frac{1}{1-v}\left(2-v-\frac{1}{1-R^{2}}\right)+\frac{1}{2\left(1-v\right)}\left(-4+2v+\frac{3}{1-R^{2}}\right)g\\ s_{1122}=s_{2211}=\frac{1}{8\left(1-v\right)}\left(1-\frac{1}{1-R^{2}}\right)+\frac{1}{16\left(1-v\right)}\left[-4\left(1-2v\right)+\frac{3}{1-R^{2}}\right]g\\ s_{1133}=s_{2233}=\frac{R^{2}}{2\left(1-v\right)\left(1-R^{2}\right)}-\frac{1}{4\left(1-v\right)}\left(1-2v+\frac{3}{1-R^{2}}\right)g\\ s_{3311}=s_{3322}=\frac{1}{2\left(1-v\right)}\left[-\left(1-2v\right)+\frac{1}{1-R^{2}}\right]+\frac{1}{4\left(1-v\right)}\left[2\left(1-2v\right)-\frac{3}{1-R^{2}}\right]g\\ s_{1212}=-\frac{R^{2}}{8\left(1-v\right)\left(1-R^{2}\right)}+\frac{1}{16\left(1-v\right)}\left[4\left(1-2v\right)+\frac{3}{1-R^{2}}\right]g\\ s_{1313}=s_{2323}=\frac{1}{4\left(1-v\right)}\left(1-2v+\frac{1+R^{2}}{1-R^{2}}\right)-\frac{1}{8\left(1-v\right)}\left(1-2v+3\frac{1+R^{2}}{1-R^{2}}\right)g \end{array}$$

When R<1, the expression is

(A6) $$g=\frac{R}{{\left(1-R^{2}\right)}^{3/2}}\left[\arccos R-R{\left(1-R^{2}\right)}^{1/2}\right]$$

When R>1, the expression is

(A7) $$g=\frac{R}{{\left(1-R^{2}\right)}^{3/2}}\left[R{\left(1-R^{2}\right)}^{1/2}-\arccos R\right]$$

When R=1, the expression is

(A8) $$\begin{array}{c} s_{1111}=s_{2222}=s_{3333}=\frac{7-5v}{15\left(1-v\right)}\\ s_{1212}=s_{2323}=s_{3131}=\frac{4-5v}{15\left(1-v\right)}\\ s_{1122}=s_{2233}=s_{3311}=s_{1133}=s_{2211}=s_{3322}=\frac{5v-1}{15\left(1-v\right)} \end{array}$$
